# Lecture on Pulsatilla

**Published:** 1885-06

**Authors:** J. T. Kent

**Affiliations:** St. Louis


### LECTURE ON PULSATILLA.

PROFESSOR J. T. KENT, M. D., ST. LOUIS.

CHARACTERISTICS: The symptoms which Pulsatilla produces are best developed in the female. It will, of course, cure complaints developed in the male, but it is very largely endowed with symptoms peculiar to women. The Puls. patient, when she begins relating her case, will weep; if your office is warm, she will become uneasy, nervous, and greatly disturbed; if there is a window open, she will go to it and sit down; she becomes dizzy in the warm room; relieved in the open air—even chilliness is relieved in the open air; she has pains flying from one part to another. Another condition is: Sour stomach, mouth, and eructation; she does not vomit, but the warm room aggravates her; veins full and engorged; puffiness. There may be swelling and burning of the ankles and top of the feet, but not in the soles.

The characteristic pain of Puls. is that of a tension, which increases until it becomes very acute, and then lets up with a snap; sensation of throbbing and pulsation throughout the body which is of a nervous character. In this blueness and engorgement of the veins, Pulsatilla is similar to Hamamelis and Zincum; glands swollen, painful, and hot; the itching of Pulsatilla is made neither better nor worse by scratching. (Sulphur is relieved by scratching.)

A peculiarity of Puls. is the changing of its symptoms. Menses intermit; the stools change—no two alike; the pains change from place to place, the itching from spot to spot. Above all, the mental states change quickly and the patient is irresolute and changeable.

MIND: Patient imagines a naked man is wrapped up in the bedclothes with her; both sexes dread each other; religious melancholy; forgetful during lucid moments. This dread of men comes on in a peculiar state of the mind, attended with disorders of menstruation and genital organs. (Aurum and Psorinum also have religious melancholy; Lachesis and Lilium tig. have it secondarily; Lycopodium, also, has great religious melancholy.) The mental state of Puls. is aggravated in evening, the stomach symptoms in the morning; patient is mild, tearful, gentle, and yielding. If she has blonde hair and blue eyes, Puls. will act quicker than if she is a brunette; but you must not think that it will not cure brunettes also.

Pulsatilla acts well with opium-eaters. Cyclamen is very similar to Puls. in female complaints, but it is worse in the fresh air and better in the warm room. The headaches are also distinguishing: The Puls. is a frontal headache; Cyclamen has generally a semi-lateral headache.

HEAD: Puls has a great many headaches connected with menstrual and gastric disorders—headaches which come and go; they gradually increase until they become very violent; worse in the warm room and in the evening; better in the open air; she kicks the covers off; she is warmer with a little covering than with a great deal.

EYE: Puls. is a great eye remedy. Feeling of blindness in connection with vertigo or suppression of the menses; everything turns dark before the eyes; subject to styes, especially on the upper lids (also Staph., Thuja, and Graphites); the Puls. patient loves to be bathed. The eye-symptoms of Puls. are most generally connected with some trouble of the genital organs.

Euphrasia, Dulc., Sulph., and Puls. are similar in symptoms of the eyes, nose, bronchi, and skin. Puls. is like Cham. in a general way, but is distinguished by its mental state (Aconite also).

Ear: Discharges from the ear, with deafness.

The discharges of Puls. are mild (like its mental state), with the exception of the leucorrhœa, which is acrid. In these conditions Puls. will improve your patient for a long time, and then must be followed by its chronic complementary remedies. Some ladies have abscesses form in their ears every time they menstruate. If Puls. does not completely cure, follow with Silicea. Puls. patient is always burning with general bodily heat, always too warm. (When there is that overheated condition of the body and the feet get cold, Silicea.) In servant girls, complaints brought on from working over hot stoves.

Nose: Loss of smell, with catarrh; large, green, fetid scales in the nose. (If the bone itself is diseased and there is a green, fetid accumulation, Aurum.) The Puls. patient has no trouble to keep warm; gets into a draft voluntarily and gets a cold without being aware of it. (It is just the reverse with Calcarea.)

Face: Alternately pale and red—usually red; eyes sunken; nose and cheeks pale; she has a sensation of flushes in the face, of intense warmth all over the body. Sometimes they feel as if a warm breeze was blowing over them, and then they shudder; there is alternate chilliness and heat.

Puls. has an eruption about the face that begins upon the tragus; the face may be red, dusky, mottled, or dark red. Erysipelas of the leg may become dusky, but you need not change your remedy on that account.

Fevers: Corresponds to the eruption of measles very nicely. (Euphrasia, Aconite, and Sulph. also. Distinguish Aconite by its *mental state.* The Euphrasia rash is similar to Puls., but its discharges are acrid and burning, producing red spots.)

Cham. may be indicated by its mental state: Child is cross; wants to be carried all the time. Puls. is made worse by rapid motion; motion must be gentle (like its mental state).

Teeth: Toothache of a drawing, stretching character; made worse by application of cold water or from anything warm in the mouth; toothache liable to come on in the warm room or from warm bed; better in the open air; the patient becomes nervous, fidgety, and faints in a warm room.

Mouth: Loss of taste, with catarrh; there is a foul taste in the mouth in the morning; stomach and mouth symptoms are aggravated in the morning after awakening; foul, clammy taste—wants to rinse the mouth to clean it.

Tongue: Puls. has a white tongue. The tongue, mouth, and

18

throat are dry, but has no thirst; but there is an exception: whenever there is fever, there may be thirst. In ague, thirst during hot stage, none during cold stage, and scarcely any during the sweat. It may be either for large or small quantities.

Desires and Aversions: Aversion to fat food; stomach deranged from eating fat food. Dislikes butter, bread, and milk. (Natrum-mur. is usually the remedy for aversion to bread.) (Lyc. has a peculiar aversion to black or brown bread.)

Stomach: Puls. retards digestion when taken for some time. Eructations tasting of fat, or, when changed, taste sour. Sour eructations and vomit; sometimes vomiting of food; eructations sour, bitter, and rancid. (Nux, Ign., and Sulph. are similar to Puls. in some of their digestive symptoms.) Gastric catarrh, aggravation from warm victuals, amelioration and also aggravation from warm drinks (Phos-acid also). Getting sick from eating ice-cream: after this he craves cold things, but as soon as they become warm in the stomach, he becomes uneasy (he vomits, Phos.); Puls., a sensation of a weight in the stomach, as from a stone, early in the morning (Bry. and Nux-v.). A sensation of fullness over stomach and abdomen, caused by an accumulation of gas; can hardly get clothing together; comes on in the morning and evening. Flatulence is a characteristic of Puls., but it is more prominent of Arg-n., China, Carbo-veg., and Lyc.

Stool: No two stools alike; at one time sour, then green, and then again bloody; one stool may be fetid, another scentless; one containing fecal matter, another blood.

Urine: Dribbling of urine; little girls wet the bed. (If child wets the bed soon after falling asleep, Sepia.) Frequent, almost ineffectual, urging to urinate, with desire to draw in the abdomen.

Sexual Organs, male: Swelled testicles, following suppressed gonorrhœa. Very excellent remedy for gonorrhœa with thick, heavy, yellow-green discharge.

Female: Menses too late and too scanty; they may be of light color, or dark and black; a mere stain, or may be black and tar-like; menses suppressed or flow intermitting. (Kreosote, Mag-carb., and Cactus have intermitting menses also.) Suppression from getting feet wet (menstruation stopped from getting head wet, Bell.). The complaints of Bell. go down, those of Puls. up. In ailments coming on from getting both the head and feet wet, give Bell., because it meets the acute stage better than Puls. Puls. has violent contracting pains. Puls. and Bell. both have tension in the muscles. Puls. is a great remedy in preparing a lady for confinement (or it may be either Sepia, Culoph., Helonias, or Viburnum). Nux-vom. resembles Puls. in some respects, but it has menses too soon and lasting too long, and irritability of mind. The aggravation of Sepia is before the menses, Puls. during or after. Kreosote has a leucorrhœa following the menses—burning, causing the labia to swell and itch, and corroding the thighs. The discharge smells like "green corn." Borax has a leucorrhœa that comes on anywhere between the menses, attended with burning and swelling of the labia, burning between the thighs. Little girls sometimes have discharges of milk at puberty, lumps in the breasts before the menses, and escaping of milk-like fluid.

Pregnancy: After-pains too long and too violent. If you prepare your patient for confinement you will never have retained placenta. The cause of retained placenta is improper contraction of the uterus. Sepia and Puls. both have these irregular contractions prominently, but more especially Sepia, I believe. After weaning, Puls. is an admirable remedy to dry up the milk; useful after parturition, when a lady does not recover well and has sour stomach, etc. (Arnica is a great remedy for after-pains.) Every time the mother puts the child to the breast, there are cramps in the stomach, bowels, or uterus (Puls. and Cham.). Spasmodic pain in the small of the back, brought about every time the breast is handled (Cham. and Puls.). Every time the child nurses there is a discharge from the vagina (Silica)—not like the symptom of Calcarea: "Nursing women have their menses." When the breasts are active, the ovaries should be dormant. Every time the child nurses there is a violent pain shooting from the nipple through to the back (Croton-tig.).

Chest: Oppression of the chest on walking fast. (Ars. patient desires to move fast.) Loose, teasing cough in the morning, with yellow expectoration; but it is a dry cough in the evening. Puls. patient is worse after lying down and getting warm in bed. Every time the wood-sawer gets warmed up he gets a cough. In some forms of phthisis it is a good remedy. Puls. is a one-sided remedy in many of its complaints, and it may be either side. It may have a sweat or a chill on one side, or chill on one arm or leg, followed by sweat on the same or opposite side.

Heart: In some affections of the heart the patient will be taken suddenly with nervous pains in the heart while walking; this is very prominent. It even relieves organic disease of the heart; that is, it enables the patient to live more comfortably.

LIMBS: Useful in curvature of the spine in little girls. Pains in the rheumatic state of Puls. shoot from one place to another; the swelling of the joints will change about, first appearing in the wrist, then in the elbow, and then in the knee, having no permanent site; attended by redness and swelling and improved by cold applications. When, in rheumatism of feet, cold water relieves, it is a distinguishing feature of Puls. (Ledum also has relief from ice-cold water, the trouble being confined to the feet, the legs being swelled and bloated.) A Ledum case: Patient is a toper, perhaps has had syphilis; deep bone pains; inflammation of the feet, attended with dropsy, heat, redness, and swelling; likes to stick feet in ice-water, and gets relief therefrom.

SKIN: There may be an eruption, with a very hyperæsthetic skin; she will shrink when you touch it. The skin is glossy, red, and looks inflamed. She says it itches and burns, but cannot bear touch; nervous and excited; sometimes relieved by cold; aggravated from any one walking over the floor. (The Apis eruption is relieved by washing in cold water.) Ulcerations that come upon the leg, chronic or acute, inflammation extending far around; the edges are elevated, dark, dusky, and tumid. The surrounding skin becomes dark and dusky. Puls. is a great remedy for dark varicose veins. Diseased limb (especially if it be the lower) becomes withered; it is not paralyzed, but atrophied; the more pain, the greater the chilliness. Chilblains that burn, smart, are blue, tender, and sore to the touch.

NERVES: Puls. is a great nervous remedy, connected with gastric and catarrhal states and the genital organs in women. There is a tired, worn-out feeling. Puls. has as much as Ars. the tired feeling. She can scarcely drag around; still, the uneasiness compels her to move, though not relieved by so doing.

SLEEP: Restless sleep, disturbed by bad dreams, sexual and unpleasant; talks and cries in sleep; dreams a black pall visits her (also Opium).

Chill, heat, etc. (Apis, Arnica, and Ars. have thirst during the chill). The Puls. chill is likely to be associated with some uterine disorder; or patient has been a high liver; heat of the face or of one hand, with coldness of the other; body hot, limbs cold. Puls. has pains with chilliness during the sweat, yet is not cold to the touch. During apyrexia, headache, mucus diarrhœa, loss of appetite, enlarged spleen, dysenteric diarrhœa, sour vomit and stomach precede the chill, fever, and sweat, which come on the next day, in which the pain commences in the evening and grows worse before midnight.

If child has had too much Cham., give it Puls; if too much Puls., give it Cham. Measles, even with typhoid symptoms, eruptions, torpid earache, short dry or loose cough, which are prone to remain as sequlæ. If cough remains after measles and Puls., Euphrasia, and Sulph. have failed, Drosera is likely to be indicated, especially if it is a dry, teasing cough, aggravated every time the child's head touches the pillow. Apis and Puls. both have better from washing and in the open air, but the Apis patient is snappish and jealous (Hyos. also), while the Puls. patient is mild, gentle, and tearful.

